# Symptomatology, prognosis and clinical findings of STEMI as a ramification of COVID-19: A systematic review and proportion meta-analysis^☆^

**DOI:** 10.1016/j.amsu.2022.103429

**Published:** 2022-03-08

**Authors:** Vikash Jaiswal, Talal Almas, Song Peng Ang, David Song, Nishat Shama, Tatyana Storozhenko, Kriti Lnu, Garima Parmar, Saria Qaiser, Sidra Naz, Akash Jaiswal, Jahanzeb Malik

**Affiliations:** a AMA School of Medicine, Makati, Philippines; b Royal College of Surgeons in Ireland, Dublin, Ireland; c International Medical University, Malaysia; d Icahn School of Medicine at Mount Sinai, NY, USA; e Bangladesh Institute of Research and Rehabilitation in Diabetes, Endocrine and Metabolic Disorders, Dhaka, Bangladesh; f Government Institution “L.T. Malaya Therapy National Institute NAMSU,” Kharkiv, Ukraine; g Department of Internal Medicine, UPMC Harrisburg, USA; h Kasturba Medical College Mangalore, India; i Rawalpindi Medical University, Punjab, Pakistan; j Harvard Medical School, Boston, MA, USA; k Department of Geriatric Medicine, All India Institute of Medical Science, New Delhi, India; l Department of Cardiology, Rawalpindi Institute of Cardiology, Rawalpindi, Pakistan

**Keywords:** COVID-19, ST-Segment myocardial infarction, Mortality, Cardiovascular disease

## Abstract

**Background:**

There is an increasing COVID-19 population with concurrent STEMI. SARS-CoV-2 poses a significant risk of hypercoagulable and/or prothrombotic events due to the disturbance in hemostasis by affecting all three components of the Virchow's triad. These abnormalities in hemostasis are an increased risk factor for cardiovascular events, including acute thrombotic occlusion of coronary arteries leading to myocardial infarction.

**Objective:**

The objective of this study is to collate the prognosis, symptomatology and clinical findings of COVID-19 adverse events causing STEMI.

**Methods:**

Databases were queried with various keyword combinations to find applicable articles. Cardiovascular risk factors, symptomatology, mortality and rates of PCI were analyzed using random-effect model.

**Results:**

15 studies with a total of 379 patients were included in the final analysis. Mean age of patients was 62.82 ± 36.01, with a male predominance (72%, n = 274). Hypertension, dyslipidemia and diabetes mellitus were the most common cardiovascular risk factors among these patients, with a pooled proportion of 72%, 59% and 40% respectively. Dyspnea (61%, n = 131) was the most frequent presenting symptom, followed by chest pain (60%, n = 101) and fever (56%, n = 104). 62% of the patients had obstructive CAD during coronary angiography. The primary reperfusion method used in the majority of cases was percutaneous coronary intervention (64%, n = 124). Mortality, which is the primary outcome in our study, was relatively high, with a rate of 34% across studies.

**Conclusion:**

Our findings show that most cases have been found in males, while the most common risk factors were Hypertension and Diabetes Mellitus. In most COVID-19 cases with ST-segment myocardial infarction, most hospitalized patients underwent primary percutaneous coronary intervention instead of fibrinolysis. The in-hospital mortality was significantly higher, making this report significant. As the sample size and reported study are considerably less, it warrants a further large-scale investigation to generalize it.

## Highlights

1) There is an increasing COVID-19 population with concurrent STEMI. SARS-CoV-2 poses a significant risk of hypercoagulable and/or prothrombotic events due to the disturbance in hemostasis by affecting all three components of the Virchow's triad.
2) Our findings show that most cases have been found in males, while the most common risk factors were Hypertension and Diabetes Mellitus.
3) The in-hospital mortality was significantly higher, making this report significant.

## 1 Introduction

COVID-19 infection continues to have a significant healthcare impact all over the world. The disease process has long evolved beyond the extrapulmonary symptomatology. Cardiovascular related diseases continue to have a significant corner in patients with concomitant COVID-19 infection.

Since the beginning of the pandemic, a wide spectrum of myocardial injury patterns have been associated with increasing cardiovascular morbidity and mortality [1,2]. These have been associated with direct myocardial injury as well as indirect effect on myocardium through stress induced injury [3]. Giustino et al. showed the different EKG and echocardiogram features that were associated with myocardial injury in patients presenting with COVID 19 [1]. One of the blind spots that continues to remain is patients with COVID-19 infection who develop ST elevation myocardial infarction (STEMI). The pathogenesis of arterial thrombosis has been associated with widespread inflammatory response causing cytokine storm, activation of coagulation cascade, plaque rupture and endothelial dysfunction as well as a mismatch in oxygen demand and supply [[4], [5], [6]]. However, there are two paradoxes associated with COVID-19 patients who develop STEMI. Literature has shown that patients who underwent percutaneous coronary intervention (PCI) had less likelihood of having obstructive coronary artery disease (CAD), revealing that sudden plaque rupture seems to be a significant potential pathophysiology [[5], [6], [7]]. Patients who have undergone PCI have had less culprit lesions that could be found that were amenable to getting stents. On the other hand, multiple studies have also shown that patients who underwent PCI for STEMI with simultaneous COVID-19 infection had higher multivessel thrombosis and stent thrombosis [4,5]. Nägele et al. also saw a higher coronary thrombus burden in patients who underwent PCI for STEMI with concurrent COVID-19 infection [8]. This suggests that the pathophysiology and the exact mechanism is not well understood.

Patients with concomitant COVID-19 and cardiovascular symptoms may have a wide presentation and STEMI may actually be the initial presentation [3,7,9]. Irrespective of the patient's initial presentation and PCI findings, patients with COVID 19 and STEMI had extended length of stay and more need for critical care, revealing that COVID 19 patients had higher STEMI associated morbidity and mortality [7].

Our aim through this proportion meta-analysis is to find the symptoms, risk factors, clinical findings, and outcomes in patient of COVID-19 having STEMI.

## 2 Methods

This systematic review was conducted and reported following the. Cochrane and PRISMA (Preferred reporting items for systematic review and Meta-analysis) 2020 guidelines [49]. The pre-specified study protocol has been registered in the PROSPERO (**CRD42021277958**). The current study is noted to be well-compliant with the AMSTAR 2 guidelines, with the quality of the present systematic review noted to be high in line with the guidelines [10].

### 2.1 Search strategy

A systematic literature search of electronic databases (PubMed, Science Direct, Cochrane Library, and Google Scholar) for peer-reviewed articles conducted in humans and published in the English language from inception up until November 2021. Boolean logic was used for conducting a database search, and Boolean search operators “AND” and “OR” were used to link search terms. The following search terms were used: “SARS-CoV-2” OR “COVID-19 “AND “ST-elevation myocardial infarction” OR “ST-segment elevation” OR “myocardial infarction” OR“acute coronary syndrome. We also screened all primary articles bibliography for additional cases. We limited our search to articles written in the English language.

### 2.2 Study selection

We included studies with a history of COVID infection followed by STEMI. The studies were carefully screened and exported to the endnote reference library software (Clarivate) and all the duplicates were removed. A manual check was carried through to crosscheck for any remaining duplicates. All studies published in English with open access were included for the review. A total of 670 reports were extracted in the initial screening. Two reviewers (VJ and NS) reviewed the papers based on title, keywords, and abstract. They (VJ and AJ) closely reviewed the articles that passed the initial screening to regulate their aptness for inclusion in the systematic analysis.Discrepancies regarding inclusion of studies were arbitrated by the senior author. Studies were also screened by backward snowballing, wherein the reference of included studies were used to guide searches.

### 2.3 Inclusion criteria

1) All studies with patients of age ≥18 along with diagnosed with COVID-19 by reverse transcription-polymerase chain reaction (RT-PCR) test
2) Patients with STEMI on electrocardiogram (ECG) at the time of admission or during hospitalization
3) All studies that described at least one of the following information regarding its clinical features, demographic, management, or outcomes of COVID-19 patients with ST-segment elevation
4) Studies such as case series, cross-sectional, multicenter and cohort studies will be included

### 2.4 Exclusion criteria

1. We will exclude data on animal studies, abstracts, editorials, commentaries, systematic reviews, Single patient case study, Letters and study with insufficient data and confirmed diagnosis of STEMI.
2. Studies done on animals or with insignificant data.

### 2.5 Data extraction and analysis

All the included articles are extracted for the following data: Study type, Author, Country, Number of patients, Age, years, Sex, Comorbidities, COVID-19 diagnosis, Symptoms, ST-elevation localization, Timing of ST-elevation post COVID infection, Echocardiogram, Ejection fraction%, RWMA, Troponin, NT pro BNP, Inflammatory biomarkers, Coronary angiography, Management Option, Diagnosis, Fibrinolysis, Coronary intervention and Outcome**.**

As this is a proportional, single-arm meta-analysis, the study estimates were first logit-transformed to normalize the data. The pooled prevalence for each variable was then obtained using a random-effect model. To quantify the amount of between-study heterogeneity, Cochran's Q statistics and Higgins and Thompson's I^2^ statistics were implemented in this study. Thereafter, if a pooled proportion of a variable were found to have high heterogeneity, sensitivity analysis, whereby each study will be removed sequentially, will be carried out to assess the robustness of the result. Publication bias was assessed using funnel plots and Egger's Regression Test. All analyses were conducted using the software R version 4.1.2 (www.r-project.org). Importantly, the present work has been reported in line with the PRISMA guidelines [11].

## 3 Results

### 3.1 Study selection

**Fig. 1:**
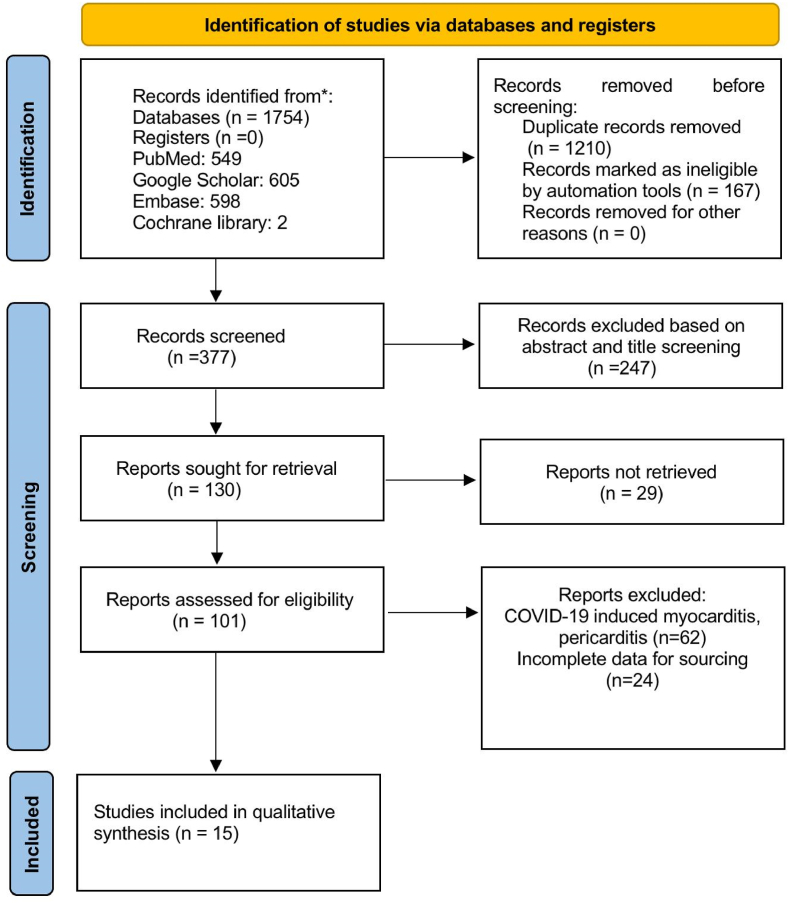
The preferred reporting items for systematic reviews and meta-analyses (PRISMA).

Preliminary database search using keywords stated above yielded 1754 articles, of which 1210 studies were excluded after removal of duplicates. 247 studies were further excluded after initial title and abstract screening. Full-text review was conducted for the remaining of 130 articles identified. A total of 15 studies that met the eligibility criteria were included in our meta-analysis. Among the 15 studies, 10 studies were case series whereas 5 studies were cohort studies in design (see Fig. 1).

### 3.2 Patient demographics, comorbidities and risk factors

**Fig. 2:**
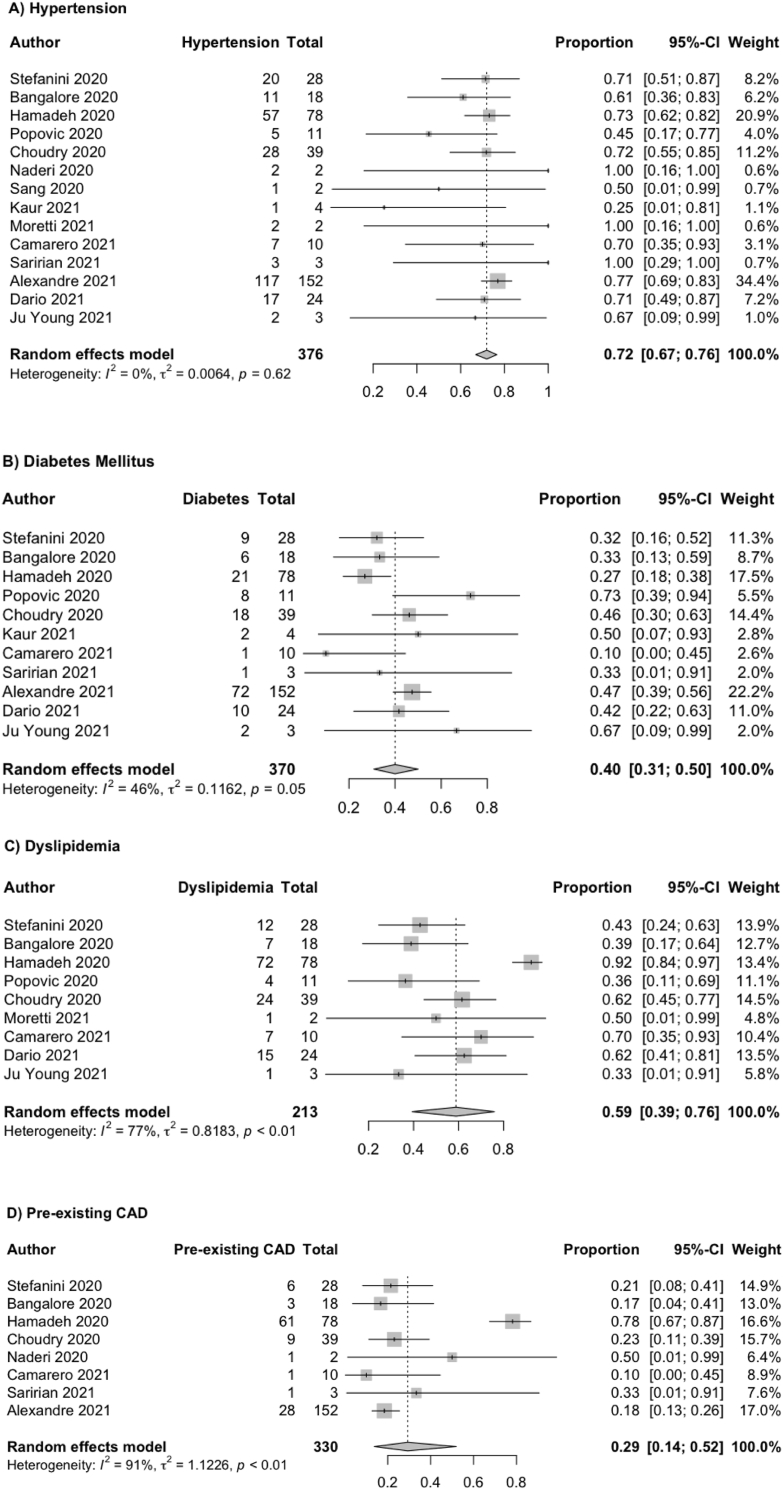
Forest plots showing meta-analysis of selected variables. **(A)** Hypertension; **(B)** Diabetes Mellitus; **(C)** Dyslipidemia; and **(D)** Pre-existing CAD.

The principal characteristics of patients were presented and summarized in Supplementary Table 1 [[36], [37], [38], [39], [40], [41], [42], [43], [44], [45], [46], [47], [48]]. The total number of patients was 379. The mean age of patients was 62.82 ± 36.01. Among the 379 patients, the majority of them are male with a pooled prevalence of 72% (95% CI: 66%–78%). Out of 15 studies, data on comorbidities were reported variably. The most common comorbidity was hypertension (72%, 95% CI: 67–76%, n = 273/376), followed by dyslipidemia (59%, 95% CI: 39–76%, n = 143/213), diabetes mellitus (40%, 95% CI: 31–50%, n = 150/370) and pre-existing CAD (29%, 95% CI 14–52%, n = 110/330). The results of the pooled prevalence of hypertension, dyslipidemia, diabetes mellitus and pre-existing CAD were illustrated in Fig. 2 respectively. In terms of heterogeneity, dyslipidemia (heterogeneity test: Q = 34.07, I^2^ = 76.5%), and pre-existing coronary artery diseases (heterogeneity test: Q = 73.93, I^2^ = 90.5%) were high. Eight out of 15 studies that reported smoking as one of the cardiovascular risk factors of CAD, 39% of the patients were found to be smokers (n = 107/277, 95% CI: 26%–54%). ECG was the initial test to perform after the onset of symptom and most common findings included ST-segment elevation in inferior leads [n = 40(35%)], anterior leads [n = 49(52%)], lateral leads [n = 14 (13%)], anterolateral lead [n = 13(33%)], and inferolateral lead [n = 4(57%)].

### 3.3 Clinical features, laboratory and echocardiographic findings

The data on clinical features, laboratory and echocardiographic findings were variably reported across studies and were summarized in Table 2 (see Table 1). Dyspnea, chest pain and fever were the most common symptoms that were reported. Pooled prevalence for dyspnea, chest pain and fever were 61% (95% CI: 47–73%), 60% (95% CI: 42–76%) and 56% (95% CI: 40%–70%) respectively. Laboratory markers included data on peak troponin, C-reactive protein (CRP) and d-dimers were synthesized and analyzed. The mean value of peak troponin, CRP and d-dimer were 70.85 ng/ml (95% CI: 3.00–1672.98), 7.09 mg/dL (95% CI: 7.05–7.13) and 1988.42 (95% CI: 1180.05–3350.56) respectively. Echocardiographic findings from 9 studies reported the mean value of left ventricular ejection fraction (LVEF) was 43.52 ± 28.24; regional wall motion abnormality was present 46 cases (65%), and 10 cases (24.40%) did not have any abnormality.

**Table 1:** Baseline demographics, comorbidities, and characteristic of included studies in the meta-analysis.

Variable	Stefanini et al.	Bangalore et al.	Hamadeh et al.	Popovic et al	Choudhary et al	Naderi et al	Sang et al.	Kaur et al.	Moretti et al.	Camarero et al.	Wong et al.	Saririan et al.
Sample (n)	28	18	78	11	39	2	2	4	2	10	3	3
Age, y(Mean)	68	63.25	64.75	63.6	61.7	58	53	60.25	68.5	67.5	41.33	63
Male, n	20	15	49	7	33	2	2	4	1	8	3	1
COVID-19 Test	RT-PCR	RT-PCR	RT-PCR	RT-PCR	RT-PCR	RT-PCR	RT-PCR	RT-PCR	RT-PCR	RT-PCR	RT-PCR	RT-PCR
**Comorbidities**												
HTN, n	20	11	57	5	28	2	1	1	2	7	–	3
HLD, n	12	7	72	4	24	–	–	–	1	7	–	–
DM, n	9	6	21	8	18	–	–	2	–	1	–	1
**Symptoms**												
Chest Pain, n	22	6	–	–	–	2	1	3	2	–	2	1
Fever, n	–	13	–	–	–	2	1	4	–	7	–	1
Dyspnea, n	19	15	–	–	–	2	1	4	1	8	–	1
**Localization of MI and Type**												
Anterior, n	4	3	–	–	24	1	–	–	1	–	3	1
Inferior, n	11	8	–	–	11	–	1	–	1	–	–	–
Lateral, n	1	9	–	–	3	–	–	–	–	–	–	–
Obstructive, n	17	8	18	11	33	2	1	–	0	9	3	0
Non-Obstructive, n	11	10	–	–	–	–	1	–	2	–	0	3
**Outcomes**												
Alive, n	16	–	–	–	–	2	2	1	1	5	3	1
Dead, n	11	13	9	3	7	–	0	3	1	3	0	2

**Table 2:** Meta-analysis of selected studies.

Variables	No of stuides	Sample Size	Pooled Proportion	95% CI(lower limit- Upper Limit)	Q	I^2^%
Hypertension	14	376	0.7195	0.6692–0.7648	10.86	0.0
Diabetes Mellitus	11	370	0.4002	0.3080–0.5001	18.39	45.6
Dyslipidemia	9	213	0.5887	0.3935–0.7595	34.07	76.5
Pre-existing coronary artery disease(CAd)	8	330	0.2929	0.1370–0.5196	73.93	90.5
Dyspnea	11	248	0.6105	0.4708–0.7341	19.51	48.7
Chest Pain	11	241	0.5996	0.4175–0.7578	45.87	78.2
Fever	8	215	0.5561	0.3994–0.7025	11.13	37.1
Smoking	8	277	0.3945	0.2648–0.5410	13.24	47.1
Obstructive CAD	12	370	0.6180	0.3854–0.8068	59.65	81.6
Non-Obstructive CAD	6	77	0.4812	0.3476–0.6175	4.28	0.0
PCI	12	222	0.6437	0.3870–0.8380	53.81	79.6
Fibrinolysis	3	100	0.3962	0.0040–0.9900	14.68	86.4
Mortality	12	372	0.3420	0.2202–0.4889	35.14	68.7

### 3.4 Angiographic findings

Data on obstructive CAD and non-obstructive CAD were synthesized from 12 to 6 studies respectively and the results were included in Table 2. One hundred and eighty six out of 370 patients were found to have obstructive CAD (62%, 95% CI: 0.3854–0.8068) while 38 out of 77 patients had non-obstructive CAD (48%, 95% CI: 35%–62%). There was substantial heterogeneity on the pooled proportion of obstructive CAD (Q = 59.65, I2 = 81.6%) while on the other hand, the heterogeneity for non-obstructive CAD was minimal (Q = 4.28, I2 = 0%).

### 3.5 Management

Pooled proportion of patients undergoing PCI and fibrinolysis were calculated from 12 to 3 studies respectively (Table 2). For PCI, 124 of 222 patients underwent the procedure with a pooled proportion of 64% (95% CI 39–84%). On the other hand, a total of 62 out of 100 patients were managed with fibrinolytic therapy, yielding a pooled proportion of 40% (95% CI 0.4–99%).

### 3.6 Outcomes and mortality

**Fig. 3:**
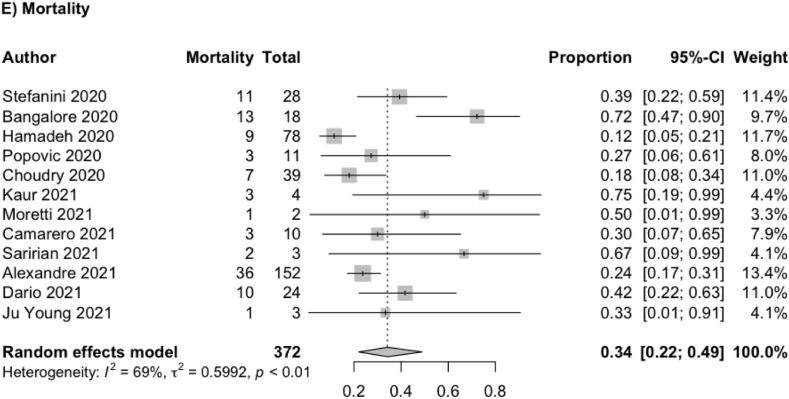
Forest plot showing meta-analysis for **(E)** Mortality.

Twelve out of 15 studies reported mortality. The pooled proportion of mortality out of a total of 370 patients was 34% (95% CI: 22%–49%) and its result was illustrated in the forest plot below (Fig. 3E). 163(70%) patients were discharged from 233 patients from the hospital.

### 3.7 Sensitivity analyses

Sensitivity analyses were carried out for the following variables with high heterogeneity (I2 >75%) for age, presence of pre-existing CAD, LVEF, and mortality. Study heterogeneity was high for age (I2 75.4%), heterogeneity dropped to 54.4% by removing Wong et al. For LVEF, the heterogeneity was 83.1% and it dropped to 58.8% by removing Kaur et al. For the presence of pre-existing CAD, the heterogeneity was 90.5% and it dropped to 0% by removing Hamadeh et al.

### 3.8 Publication bias

Evaluation of publication bias suggested presence of bias for chest pain (t = 2.413, p = 0.039) and PCI (t = 2.229, p = 0.046). Otherwise, there was no significant publication bias detected with regards to the variables tested above.

## 4 Discussion

This study documents that during the ongoing pandemic there have been significant cases of thromboembolism and MI post COVID-19 infection worldwide. Assessment of 379 COVID-19 cases with admission STEMI allowed us to reveal 186 patients with obstructive CAD and 38 patients with non-obstructive CAD. Therefore, 64% of patients underwent primary or facilitated PCI with stent implantation, while other individuals administered with optimal medical treatment. In fact, the cause of clinical symptoms might be elsewhere without functionally significant stenosis and not improved by stent implantation. Reynolds H et al. reports MI based on culprit lesion ischemic/vascular cause in 64% observed patients and 21% had an alternate, non-ischemic diagnosis, most commonly myocarditis [11]. Attending physicians followed recommendations, based on current ESC and AHA guidelines for the right clinical decision in each case [12,16,19,22].

The study population consisted mainly of males with mean age of 62.82 ± 36.01 years having a wide range of cardiovascular risk factors including smoking, hypertension, dyslipidemia, diabetes mellitus, and CAD. The presented comorbidities promote coronary lesion [13]. On the other hand, potential mechanisms of CAD could be worsened by COVID-19 infection including plaque disruption, dissection, thromboembolism, vasomotor and microvascular dysfunction, and supply/demand mismatch [14,15,24,26]. Interestingly, previous studies show that non-obstructive CAD is more commonly associated with women than men [[28], [29], [30]]. Consequently, the vast majority of people still present with MI and obstructive etiologies; simultaneously, the number of non-obstructive causes due to COVID-19 is increasing. Risk factor optimization with prompt diagnosis can reduce the progression of cardiovascular events.

As recent reports show severe cardiovascular complications are observed in a large portion of COVID-19 patients with high levels of in-hospital and long-term mortality [20,21,23,25]. Notably in our study, the mortality rate was high with 34% in all cohorts. Furthermore, elderly patients with comorbidities are associated with adverse clinical outcomes following contraction of the SARS-CoV-2 [27,31].

In addition, we found positive cardiac enzymes, clinical symptoms profile with chest pain, reduced LVEF in patients having abnormal ECG. However, it is important to recognize that non-obstructive CAD can present as obstructive CAD. Non-obstructive CAD is a working diagnosis and includes a very heterogeneous group of patients with myocarditis, Takotsubo cardiomyopathy, dilated and hypertrophic cardiomyopathy, which may also be associated with elevated troponin levels, ECG changes and wall motion abnormalities [17,18]. Moreover, COVID-19 leads to thromboembolic and disseminated intravascular coagulation disorders with high d-dimer levels [[32], [33], [34], [35]]. Therefore, proper differential diagnosis work up potentially may improve outcomes, requiring different treatments.

### 4.1 Limitations

The first limitation was a small sample size, while the study's methodology allowed us to analyze clinical features and outcomes in COVID-19 patients with STEMI. The study was retrospective, included case reports and case series, thus a selection bias could have existed. However, our analysis showed a high mortality rate and large presentations of non-obstructive CAD associated with viral infection. As we clearly demonstrated, the optimization of precise diagnostic and treatment strategies is required among these patients.

## 5 Conclusion

Our findings show that most cases have been found in males, while the most common risk factors were Hypertension and Diabetes Mellitus. In the majority of COVID-19 cases with ST-segment myocardial infarction, most hospitalized patients underwent primary percutaneous coronary intervention instead of fibrinolysis. The in-hospital mortality was significantly higher, making this report significant. As the sample size and reported study are considerably less, it warrants a further large-scale investigation to generalize it.

## Ethical approval

Obtained.

## Sources of funding

None.

## Author contribution

VJ,TA and AJ designed the study; VJ, NS, JM extracted the data; AJ, SPA, DS and NS performed the screening and selection; SPA contributed to the statistical analyses and interpretation of results; KL, VJ, DS,TA SPA, TS, and AJ drafted the manuscript, which DS, GP, SQ, SN and AJ modified and critically revised the paper. All authors read and approved the final manuscript.

## Consent

Obtained.

## Registration of research studies

1. Name of the registry: Prospero.

2. Unique Identifying number or registration ID: **CRD42021277958**).

3. Hyperlink to your specific registration (must be publicly accessible and will be checked): NA.

## Guarantor

Talal Almas

RCSI University of Medicine and Health Sciences

123 St. Stephen's Green Dublin 2, Ireland

Talalamas.almas@gmail.com

+353834212442.

## Data availability statement

All data relevant to the study are included in the article or uploaded as supplementary information.

## Statement of ethics

Not required.

## Provenance and peer review

Not commissioned, externally peer-reviewed.

## Declaration of competing interest

None.
