# Supplementary material for: Symptomatology, prognosis and clinical findings of STEMI as a ramification of COVID-19: A systematic review and proportion meta-analysis
Source: Ann Med Surg (Lond). 2022 Mar 8;76:103429. doi: 10.1016/j.amsu.2022.103429 (PMC8902059; doi:10.1016/j.amsu.2022.103429)
Supplement: Multimedia component 1 [file mmc1.docx]

**SUPPLEMENTARY FILE**

**Supplementary Table 1**: Publication Bias via Egger’s test

**Figure 2**: Funnel Plot for PCI

**Figure 1**: Funnel Plot for chest pain

| **Variables** | **Intercept** | **t value** | **p value** |
| --- | --- | --- | --- |
| **Hypertension** | 1.1876 | -1.650 | 0.125 |
| **Diabetes Mellitus** | -0.2260 | -0.297 | 0.773 |
| **Dyslipidemia** | -0.9340 | -0.480 | 0.646 |
| **Pre-existing CAD** | -0.4860 | -0.210 | 0.840 |
| **Chest Pain** | 1.9260 | 2.413 | 0.039 |
| **Fever** | 1.1690 | 2.258 | 0.065 |
| **Dyspnea** | 1.0630 | 1.954 | 0.082 |
| **Smoking** | -0.3400 | -0.433 | 0.680 |
| **Obstructive CAD** | 1.2230 | 1.195 | 0.260 |
| **Non-obstructive CAD** | 1.3370 | 2.497 | 0.067 |
| **PCI** | 2.2290 | 2.283 | 0.046 |
| **Mortality** | 1.53 | 1.704 | 0.119 |

**Supplementary Table 1**: Publication Bias via Egger’s test


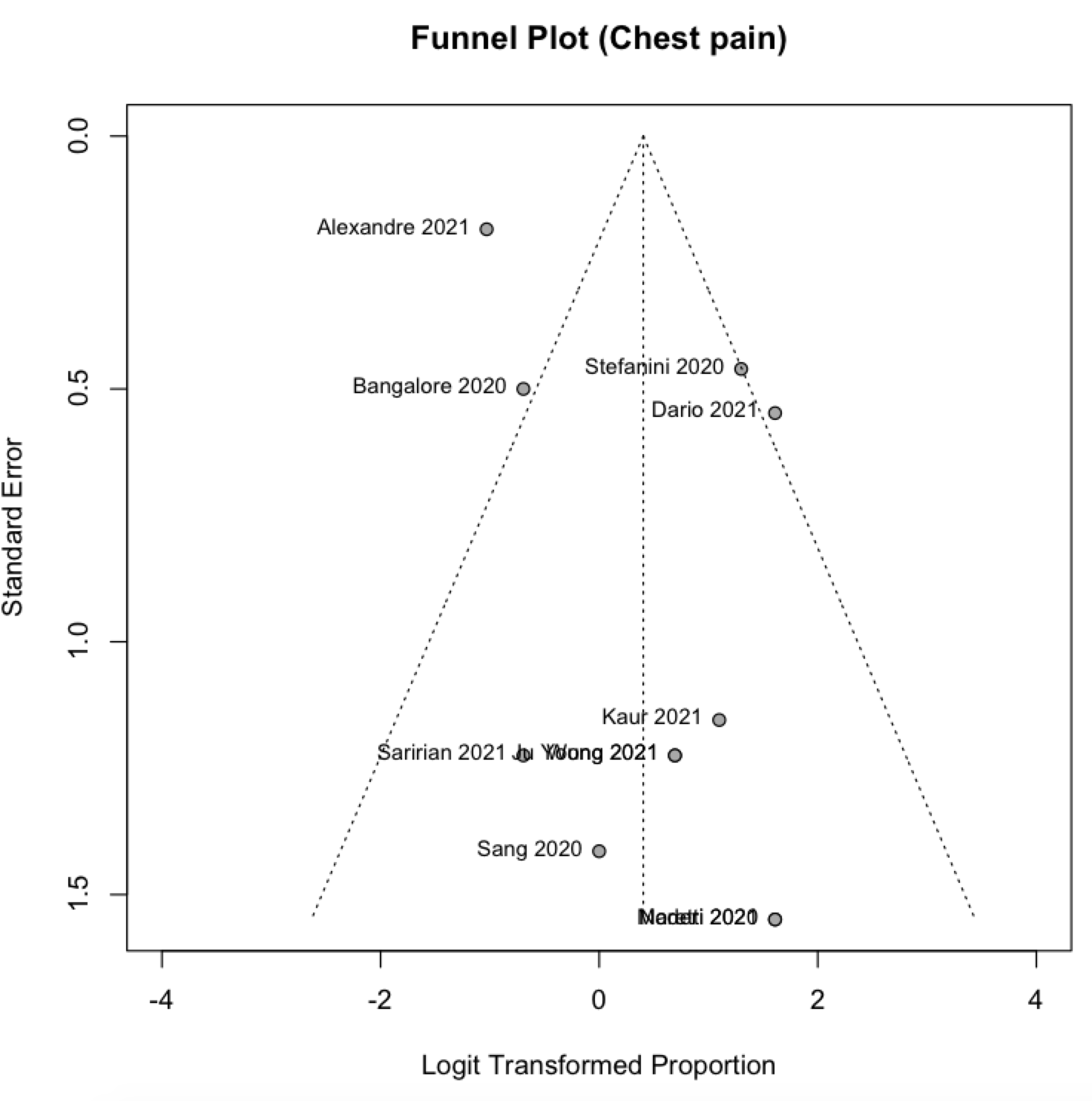

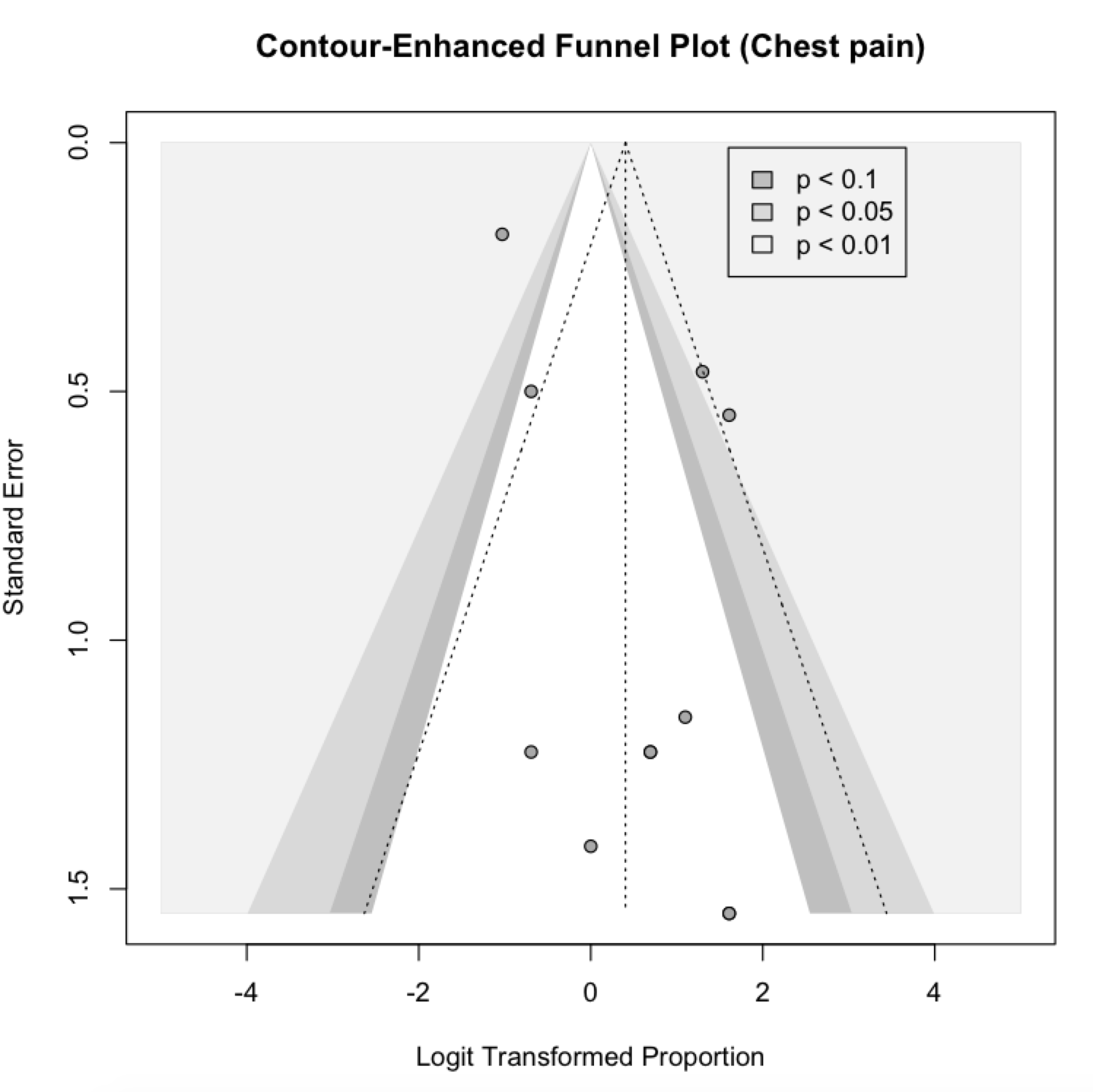


**Figure 1**: Funnel Plot for chest pain


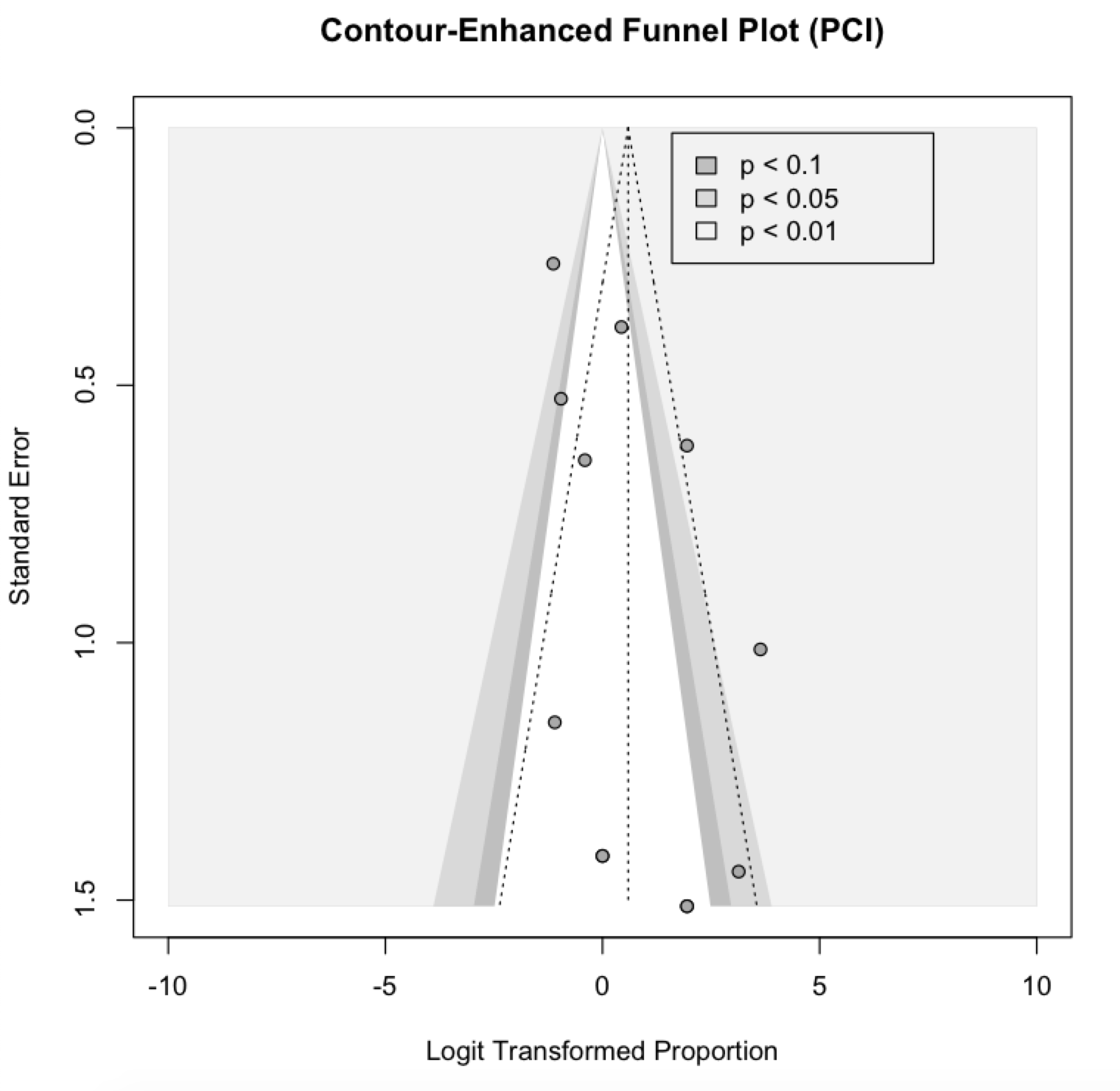


**Figure 2**: Funnel Plot for PCI
