# Supplementary material for: Symptomatology, prognosis and clinical findings of STEMI as a ramification of COVID-19: A systematic review and proportion meta-analysis
Source: Ann Med Surg (Lond). 2022 Mar 8;76:103429. doi: 10.1016/j.amsu.2022.103429 (PMC8902059; doi:10.1016/j.amsu.2022.103429)
Supplement: Multimedia component 2 [file mmc2.docx]

| **Section and Topic** | **Item #** | **Checklist item** | **Location where item is reported** |
| --- | --- | --- | --- |
| **TITLE** | | |  |
| Title | 1 | Prevalence and risk factors of psychoactive substance abuse among students in Ethiopia: A systematic review and Meta-analysis | 1 |
| **ABSTRACT** | | |  |
| Abstract | 2 | Background: Substance uses were seen in 18-25 years old age groups who are more than 40% world population, from which Africa was the region with the highest proportion accounting 60% in 2016. This review aimed to assess the burden of substance abuse among students in Ethiopia. Methods: A comprehensive search was conducted in PubMed/Medline; Science direct and African Online Journal without language and date restriction. The Heterogeneity among the included studies was checked with forest plot, χ2 test, I2 test, and the p-values. All cross-sectional studies reporting rate of prevalence of psychoactive substance among students were included and the rest were excluded. Result: A total of 545 articles were identified from different databases and 42 articles were selected for evaluation. Twenty-nine Articles with 22, 012 participants were included. The overall prevalence of psychoactive substance abuse was 32.28% (95% confidence interval (CI): 26.74 to 37.82). Conclusion: The review revealed that one-third of the high school and higher education students used different psychoactive substances in Ethiopia.  Registration: This Systematic Review and Meta-Analysis was registered in Prospero international prospective register of systemic reviews (CRD42020146656) on April 28/2020.  Keywords: psychoactive, Substance Abuse, High school, College, University | 1 |
| **INTRODUCTION** | | |  |
| Rationale | 3 | A substance abuse is a neglected health problem in developing countries which affects the younger and productive age groups who are the gear pivots of development of a nation. A number of observational studies were conducted among students in Ethiopia in different regions of the country. However, the pooled prevalence and risk factors of psychoactive substance abuse among students in Ethiopia, particularly in high school students is uncertain and a topic of debate.. | 2 |
| Objectives | 4 | This systemic review aimed to investigate the national prevalence and reasons of substance abuse among Ethiopian students | 3 |
| **METHODS** | | |  |
| Eligibility criteria | 5 | All cross-sectional studies reporting prevalence and risk factors of psychoactive substance abuse among high school, college and University students in Ethiopia without date and language restriction were included | 4 |
| Information sources | 6 | A three steps search strategy was employed in this review. An initial search on PubMed/Medline, Science direct and African online journal databases were carried out followed by an analysis of the text words contained in Title/Abstract and indexed terms. A second search was undertaken by combining free text words and indexed terms with Boolean operators. The third search was conducted with the reference lists of all identified reports and articles for additional studies. Finally, the additional and grey literature search was conducted on Google scholars up to ten pages. | 4 |
| Search strategy | 7 | The search strategy was used as follows by modifying the Mesh terms and combing Boolean operators: student OR high School student OR college student OR university student OR compass student AND substance OR alcohol OR Khat OR Catha Edulis OR Chat OR mirra OR mairungi OR Cigarrate OR shisha OR psychoactive substance OR cocaine OR opioids OR marijuana AND addiction OR abuse OR burden OR Magnitude OR prevalence. | 4 |
| Selection process | 8 | All cross-sectional studies reporting prevalence and risk factors of psychoactive substance abuse among high school, college and University students in Ethiopia without date and language restriction were included | 5 |
| Data collection process | 9 | The data from each study were extracted by SM and YA independently with Microsoft excel format and imported for analysis in R software version 3.6.1 | 5 |
| Data items | 10a | The extracted data includes: author, year of publication, event, sample size, region and design | 5 |
|  | 10b | The majority of included studies identified the possible risk factors of substance use among students including but not limited to gender, age, marital status, educational level, and occupation. | 5 |
| Study risk of bias assessment | 11 | The methodological quality of each study was evaluated with tools adopted from Joanna Briggs institute for Mata-analysis of cross-sectional studies | 5 |
| Effect measures | 12 | The effect measures were proportion, OR with 95% confidence interval | 5 |
| Synthesis methods | 13a | All cross-sectional studies conducted to assess any form of psychoactive substance use among students in Ethiopia | 5 |
|  | 13b | Articles identified for retrieval were assessed by two independent Authors for methodological quality before inclusion in the review using a standardized critical appraisal Tool adapted from the Joanna Briggs Institute . The disagreements between the Authors appraising the articles were resolved through discussion. Articles with average scores greater than fifty percent were included for data extraction | 5 |
|  | 13c | Description of included studies and the Joana Briggs appraisal tool were presented with table | 8 |
|  | 13d | We conducted the meta-analysis with random effect model with restricted maximum likely hood method because of substantial heterogeneity between the included studies. The meta-analysis was conducted in STATA version 16 software | 5 |
|  | 13e | The pooled prevalence of substance use were determined with a random effect model as there was substantial heterogeneity. The Heterogeneity among the included studies was checked with forest plot, χ2 test, I2 test, and the p-values. Substantial heterogeneity among the included studies was investigated with subgroup analysis and meta-regression | 5 |
|  | 13f | Sensitivity analysis was done to evaluate the influential studies and further analysis was made after removing the outliers. | 5 |
| Reporting bias assessment | 14 | Publication bias was checked with a funnel plot and the objective diagnostic test was conducted with Egger's correlation, Begg's regression tests, and Trim and fill method. | 5 |
| Certainty assessment | 15 | We assess the methodological quality of individual studies with Joana Briggs appraisal tool and the quality of this meta—analysis with AMSTAR 2 tool but we didn’t do as GRADEpro is not applicable for cross-sectional studies | 5 |
| **RESULTS** | | |  |
| Study selection | 16a | A total of 545 articles were identified from different databases with an initial search. Forty-two articles were selected for evaluation after the successive screening. Twenty-nine Articles with 22012 participants were included in the systematic review and Meta-Analysis | 8 |
|  | 16b | Thirteen studies were excluded with reasons | 8 |
| Study characteristics | 17 | All of the included studies were cross-sectional studies and their methodological qualities were moderate to high. The majority of studies were from the Amhara regional state followed by Oromia and SNNPR. There was no study from Afar and Benshangul Gumuz regional states. The majority of included studies were conducted in Universities (17) followed by High Schools (8) and college (4). Eighteen studies tried to assess the prevalence and associated factors of more than two substance uses, twelve studies assess only the prevalence and associated factors of Khat among students and one study assessed the prevalence of Cigarette smoking alone | 8 |
| Risk of bias in studies | 18 | Funnel plot didn’t show publication bias and rank correlation and eggers linear regression didn’t show significant publication bias | 13 |
| Results of individual studies | 19 | This systematic review and Meta-Analysis was intended to provide evidence on prevalence and risk factors of substance use among students in Ethiopia. | 9, 10,11,12 |
| Results of syntheses | 20a | Synthesis of results was carried out with the STATA 16. The Heterogeneity among the included studies was checked with forest plot, χ2 test, I2 test, and the p-values. | 5 |
|  | 20b | Described within the document | 9, 10,11,12 |
|  | 20c | Subgroup analysis were conducted | 11 |
|  | 20d | We conducted but we didn’t report the results of the sensitivity analysis as there was no significant effect on summary effects | 13 |
| Reporting biases | 21 | Risk of bias was done and reported | 13 |
| Certainty of evidence | 22 | We assess the methodological quality of individual studies with Joana Briggs appraisal tool and the quality of this meta—analysis with AMSTAR 2 tool but we didn’t do as GRADEpro is not applicable for cross-sectional studies | 5 |
| **DISCUSSION** | | |  |
| Discussion | 23a | The overall pooled prevalence of psychoactive substance abuse in this systematic review and Meta-analysis was 32% (95% confidence interval (CI): 27 to 38, 29 studies, 22, 012 participants). | 14 |
|  | 23b | The review incorporated plenty of studies with a large number of participants but the majority of studies included in this review didn’t report risk determinants for factor analysis. The included studies were conducted in a different setting, and population which caused substantial heterogeneity. Besides, there were a limited number of studies in some countries and it would be difficult to provide conclusive evidence with results pooled from a fewer study. | 15 |
|  | 23c | Body of evidence revealed that the prevalence of psychoactive substances among students is very high. It is very shocking having the prevalence of psychoactive substances among high school students who are supposed to handover and sustain the countries development. Technological innovation, discovery, and societal development are not expected if the young productive populations are suffered from psychoactive substances. Therefore, different strategies are required as early as possible to prevent substance abuse among students | 15 |
|  | 23d | The meta-analysis revealed that the prevalence of psychoactive substances among students was very high and the main reasons for substance use were identified. However, the included studies were too heterogeneous, and cross-sectional studies also don’t show a temporal relationship between mortality and its determinants. Therefore, further observational and randomized controlled trials are in demand for a specific group of patients by stratifying the possible independent predictors | 15 |
| **OTHER INFORMATION** | | |  |
| Registration and protocol | 24a | This systematic review and meta-analysis was registered in Prospero's international prospective register of systematic reviews (CRD42020146656) on April 28/2020. | 4 |
|  | 24b | This systematic review and meta-analysis was registered in Prospero's international prospective register of systematic reviews (CRD42020146656) on April 28/2020 and updated on June, 2021 | 4 |
|  | 24c | Describe and explain any amendments to information provided at registration or in the protocol. | 4 |
| Support | 25 | No funding was obtained from any organization | 17 |
| Competing interests | 26 | The authors declare that there are no competing interests | 17 |
| Availability of data, code and other materials | 27 | Data and material can be available where appropriate | 17 |
